# Identification of an immunogenic cell death-related gene signature predicts survival and sensitivity to immunotherapy in clear cell renal carcinoma

**DOI:** 10.1038/s41598-023-31493-z

**Published:** 2023-03-17

**Authors:** Shuoming Zhou, Yanwen Lu, Yuxin Chen, Weidong Gan

**Affiliations:** Department of Urology, Affiliated Drum Tower Hospital, Medical School of Nanjing University, Nanjing, China

**Keywords:** Computational biology and bioinformatics, Cancer genetics, Cancer genomics, Urological cancer

## Abstract

Immunogenic cell death (ICD) is the trigger of adaptive immune responses. However, the role of ICD-related genes in clear cell renal carcinoma (ccRCC) remains unclear. We aimed to identify biomarkers associated with ICD and develop an ICD-related predictive model that predicts the immune microenvironment, prognosis, and response to immunotherapy in ccRCC. Our study included 739 patients (603 in the training set and 136 in the validation set) with clinicopathologic information and transcriptome sequencing data. Consensus clustering, principal component analysis (PCA), weighted gene co-expression network analysis (WGCNA), univariate COX analysis, multivariate COX analysis, and the Lasso-Cox algorithm were applied to shrink predictors and construct a predictive signature of overall survival (OS). We used CIBERSORT, ESTIMATE, and TIMER in the R package IOBR to evaluate the tumor microenvironment and immune infiltration pattern of each sample. Finally, the single cell sequencing results of immune cells in ccRCC were used to verify the results of immune infiltration analysis, and the performance of the prognostic model was evaluated by calibration curves and c-index. This study revealed that inability of the initial immune response and primary immunodeficiency were significantly enriched in the ICD subgroup with poor prognosis. We found that the ten candidate ICD genes (CALR, ENTPD1, FOXP3, HSP90AA1, IFNB1, IFNG, IL6, LY96, PIK3CA, and TLR4) could affect the prognosis of ccRCC (p < 0.05). The prediction model (PRE) we constructed can not only predict the long-term survival probability but also evaluate the landscape of immune infiltration in ccRCC. Our study demonstrated that low infiltration of dendritic cells in ccRCC implies a poor prognosis, whereas the degree of CTL infiltration is less important. An individualized prediction model was created to predict the 1-, 2-, 3-, and 5-year survival and responsiveness of ccRCC patients to immunotherapy, which may serve as a potent tool for clinicians to make better treatment decisions and thus improve the overall survival (OS) of ccRCC patients in the future.

## Introduction

Clear cell renal cell carcinoma (ccRCC) is the most common pathological type of renal cell carcinoma (RCC) and is typically characterized by malignant epithelial cells with clear cytoplasm, accounting for more than 70% of RCC^1–3^. Although most limited ccRCCs are slow-growing and non-fatal, approximately 1/3 of patients present with local or distant metastases at initial diagnosis^4,5^, implying a poor prognosis. Currently, surgical resection is the treatment of first choice for ccRCC^6^. However, among ccRCC patients who are eligible for radical surgical treatment, about 1/4 will experience recurrence and metastases after surgery^7^.

Given the resistance of ccRCC to conventional adjuvant therapies such as chemotherapy and radiotherapy^8–10^, adjuvant therapy for ccRCC has largely shifted to targeted therapy and immunotherapy. Unfortunately, targeted therapies for ccRCC are often met with resistance, resulting in poor clinical outcomes. Therefore, immunotherapies such as IFN-gamma, immune checkpoint blockade, and cancer vaccines have emerged as promising treatment options for ccRCC patients^11^. Not all patients can benefit from immunotherapy to prolong survival, probably due to the differences in tumor microenvironment and susceptibility to immunotherapy in each patient.

Accumulating evidence has proved immunogenic cell death (ICD) is the trigger of adaptive immune responses through its association with danger signal pathways mediating the extracellular emission of danger signals^12–14^. In cancer immunotherapy, the key point is to harness the immune system to elicit an anti-tumor immune response. Comprehensive preclinical studies have established ICD as a potent predictor of antitumor immunity^12,15–18^. Results from a large-scale META study revealed that the ICD-related genes exhibited a highly-clustered and largely cancer type-specific prognostic impact^19^. Furthermore, this META study analyzed the prognostic role of ICD in lung, breast, and ovarian cancers. Recently, related studies have also been performed in HNSCC^20^, but to the best of our knowledge, no similar studies have been reported in ccRCC.

Previously, miRNA- and lncRNA-based gene signatures were used to diagnose RCC^21,22^, but the above models do not investigate the tumor immune microenvironment or immunotherapy responsiveness. In this study, we aimed to identify biomarkers associated with ICD and develop an ICD-related predictive model called "PRE", which predicts the immune microenvironment, prognosis, and response to immunotherapy in ccRCC.

## Materials and methods

### Selection and analysis of datasets

Download ccRCC expression data from the TCGA and ICGC databases. UCSC Xena (https://xenabrowser.net/datapages/) is a storehouse for a wide variety of public data, including the TCGA database, and has been processed for batch effect elimination, which is of high use. The high-throughput sequencing data used in the training set of this study were obtained from UCSC Xena, with the format of the sequencing data being FPKM. And the expression profile for the validation set was obtained from the ICGC (International Cancer Genome Consortium) database. The ICGC database contains 89 cancer projects from 17 administrative regions in Asia, Australia, Europe, North America and South America, including 25,000 tumor genomes, with data from several RCC cohorts, such as RT-US, RECA-CN, CCSK-US, WT-US, and RECA-EU. The data used in this study's validation set came from RECA-EU, and the sample size was greater than 100.

Row (gene) and column (sample) with NA value greater than 50% were then removed. Finally, we performed log2 (X + 1) normalization on the FPKM data.

The immunohistochemical results of PIK3CA, IL1R1, HSP90AA1, and ATG5 from renal tumors were downloaded from the Human Protein Atlas (HPA) database using the R package "HPAanalyze"^23^ and matched immunohistochemistry images and information were obtained to compare and analyze the immunohistochemistry results for these four genes.

### Prognosis-related ICD genes screening

Based on the expression profile data and the corresponding clinical data of the patients, we bulk screened for ICD genes that were significantly associated with tumor prognostic parameters such as OS. In this study, we assessed the prognostic significance of each ICD gene by the Survival package in R, integrating survival time, survival status, and gene expression data, using univariate Cox hazard analysis.

### Consensus clustering

The clustering was performed via ConsensusClusterPlus package in R^24^, using agglomerative pam clustering with a 1-pearson correlation distance and resampling 80% of the samples for 10 repetitions. In this study, the k-means clustering algorithm was used to obtain stable clustering subgroups by learning the characteristics of mRNA expression of 11 ICD genes that were significantly associated with prognosis. The optimal number of clusters was determined by using the plot of the empirical cumulative distribution function.

### Principal component analysis (PCA)

First, we use the PCA function in the Factoextra and FactoMineR packages in R to extract the features and cumulative contribution of each principal component. Then the principal components with the first and second contribution rates are selected to construct the new coordinate system, and the distribution status of subgroups in the new coordinate system is reconstructed. In accordance with the subgroup distribution status, we selected the major component associated with the target subgroup tightly and proceeded to analyze the contribution values of each original element. In the end, the principal components most relevant to the target subgroup were identified and the key genes to focus on for analysis were found.

### Weighted gene co-expression network analysis (WGCNA)

To further explore the functional characteristics of the target subgroup, this study mainly used the R package WGCNA for weighted gene co-expression network analysis^25^. We calculated the correlation of all genes with the key genes FOXP3 and LY96 obtained from principal component analysis, screened out the genes that were significantly associated with the key genes (p < 0.05), and introduced these genes into WGCNA to obtain the gene co-expression module. The scale-free R^2^ was set to greater than 0.80 and the soft-gap value was set to 6^25^. These modules were then used for correlation analysis with ICD subtypes to select the most relevant module to the target ICD subtype.

### Functional enrichment analysis and gene set enrichment analysis (GSEA)

Gene Ontology (GO) enrichment analysis and The Kyoto Encyclopedia of Genes and Genomes (KEGG) enrichment analysis were performed on the obtained modular genes using the R package ‘ClusterProfiler’^26^.

Next, we obtained the GSEA software (version 3.0) from the GSEA (http://software.broadinstitute.org/gsea/index.jsp) website and divided the samples into two groups based on the consensus clustering results. The subset (c2.cp.kegg.v7.4.symbols.gmt) was also downloaded from the Molecular Signatures Database (http://www.gsea-msigdb.org/gsea/downloads.jsp) to evaluate the relevant pathways and molecular mechanisms.

### Screening of variables included in predictive model PRE

The dimensionality reduction analysis of LASSO-COX was performed by glmnet and survival packages in R. Lastly, 10 candidate genes and corresponding values of lambda (λ) were obtained according to ccRCC patients' OS in the training set. Riskscore = b1 × 1 + b2 × 2 + ⋯ + bixi, where the bi can be obtained from the lambda value and xi is the gene expression value^27^.

### Characterization of immune landscape and prediction of response to immunotherapy

IOBR [https://doi.org/10.3389/fimmu.2021.687975] is a computational tool for immuno-oncology biology research. We used [CIBERSORT (DOI: (https://doi.org/10.1038/nmeth.3337), ESTIMATE (https://doi.org/10.1038/ncomms3612), TIMER (https://doi.org/10.1186/s13059-016-1028-7)] in the R package IOBR to evaluate the tumor microenvironment and immune infiltration pattern of each sample. Finally, we used TIMER2.0 and TISCH tools to verify the results of immune infiltration analysis^28^.

Tumor immune dysfunction and exclusion (TIDE) analysis was used to determine the responsiveness of patients to immunotherapy. TIDE (http://tide.dfci.harvard.edu/) as an analytical technique uses two major mechanisms of tumor immune evasion to predict immunotherapeutic response: T cell dysfunction and T cell suppression in tumors with low CTL levels.

### Constructing and validating a nomogram for predicting overall survival

The variables of risk score, age, T-stage, grade, and gender were first subjected to multivariate cox regression analysis. The statistically significant variables from the results of the multivariate cox analysis were then used to build the ccRCC survival prediction model, and the model results were visualized in the form of Nomogram plots. Calibration plots ware used to compare predicted and actual OS for 1-, 2-, 3-, and 5-year survival probabilities in the training and validation groups.

### Finding potential therapeutic compounds

To determine which target drugs might be useful against ccRCC cancer cells, we used the Broad Institute’s Connectivity Map build 02^29,30^, a public online tool (https://portals.broadinstitute.org/cmap/) that needs to be registered with a non-profit account that allows users to predict compounds that might activate or inhibit cancer cells based on a gene expression signature. We also used the SPIEDw web tool (http://www.spied.org.uk/)^31,32^ with an ICD-related genes expression signature to interrogate the CMAP database (https://portals.broadinstitute.org/cmap/)^30^. Lastly, we performed specific analysis within the Connectivity Map tools (https://clue.io/)^33^ to further investigate the mechanism of actions (MOA) and drug-target.

## Results

### Expression and prognosis characteristics of ICD-related genes in ccRCC

**Figure 1:**
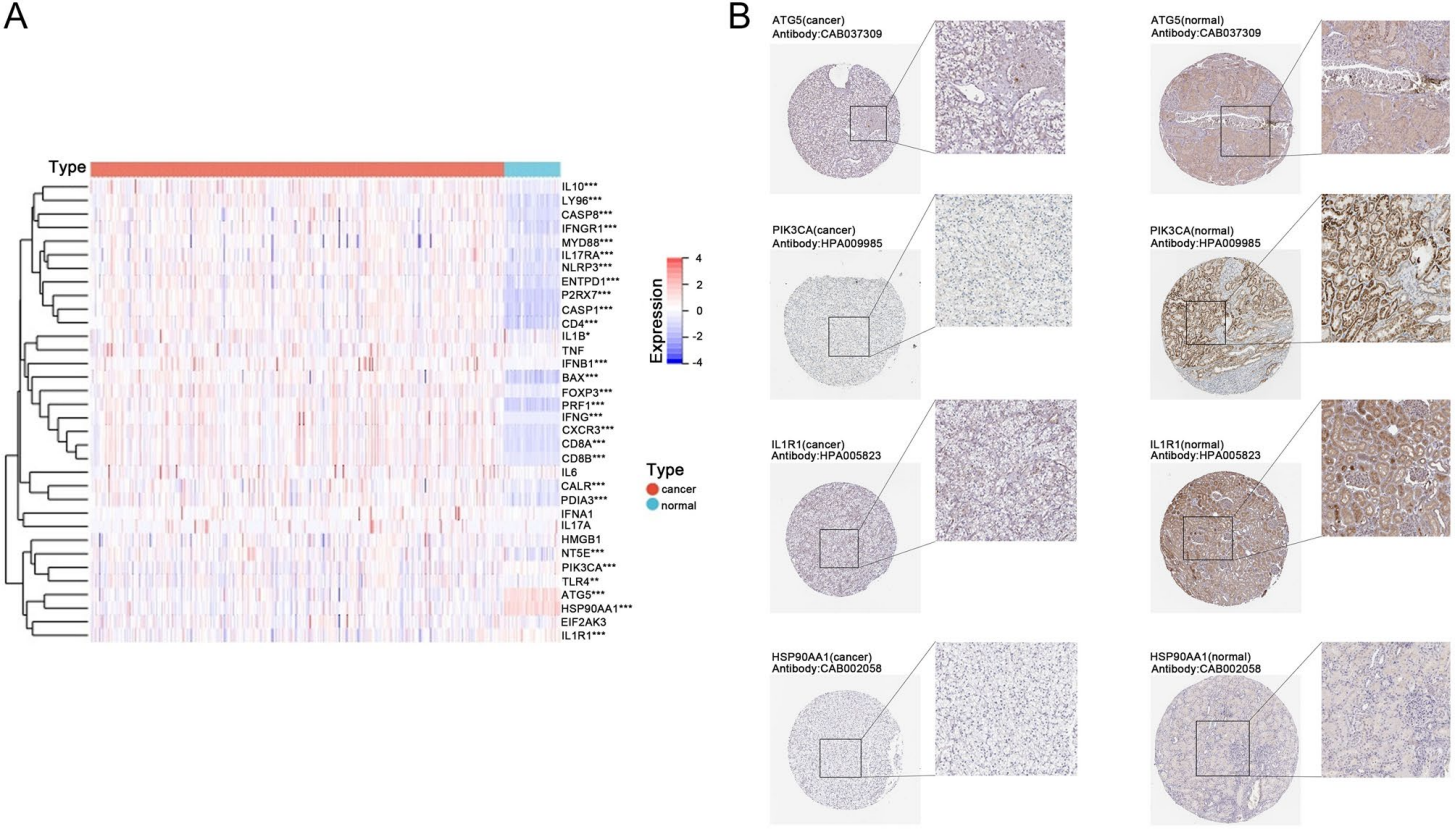
ICD genes expression profile. (**A**) Heatmap shows 34 ICD genes expression profiles of ccRCC and normal samples in TCGA database; (**B**) Immunohistochemical results of ATG5, PIK3CA, IL1R1, and HSP90AA1 in renal cancer and normal tissues; *p < 0.05; **p < 0.01; ***p < 0.001.

Based on previously published literature^19,20^, we summarized a total of 34 ICD-related genes. To explore the biological role of ICD-related genes, we first investigated whether ICD genes differ in relevant clinical features. It was found that the expression levels of 28 ICD genes were different in ccRCC tumor samples and paracancerous tissue samples in the TCGA database (Fig. 1A). Twenty-four genes were significantly highly expressed in tumor tissues (p < 0.05), including IFNG, CD8B, CXCR3, IFNB1, CD8A, P2RX7, PRF1, FOXP3, IL10, NLRP3, CASP1, CD4, LY96, ENTPD1, BAX, CASP8, IL17RA, NT5E, IL1B, IFNGR1, MYD88, TLR4, PDIA3, and CALR. PIK3CA, IL1R1, HSP90AA1, and ATG5 were significantly lower expressed in tumor tissues (p < 0.05), which were further confirmed by the protein expression profile in kidney cancer downloaded from HPA database (Fig. 1B). While the remaining six genes, TNF, IFNA1, IL17A, HMGB1, EIF2AK3 and IL6, were not significantly different between the two groups.

The univariate COX regression forest plot showed (Fig. S1) that 11 of the 28 ICD genes were significantly and statistically associated with OS (p < 0.05). Among them, IFNB1, CALR, PDIA3, FOXP3, IFNG, LY96, and IL6 high expression groups had shorter OS compared to low expression groups and were negatively correlated with survival, while this phenomenon was diametrically opposed in ENTPD1, TLR4, HSP90AA1, and PIK3CA genes.

### Consensus clustering identified two ICD-associated subtypes in the gathered ccRCC cohort

**Figure 2:**
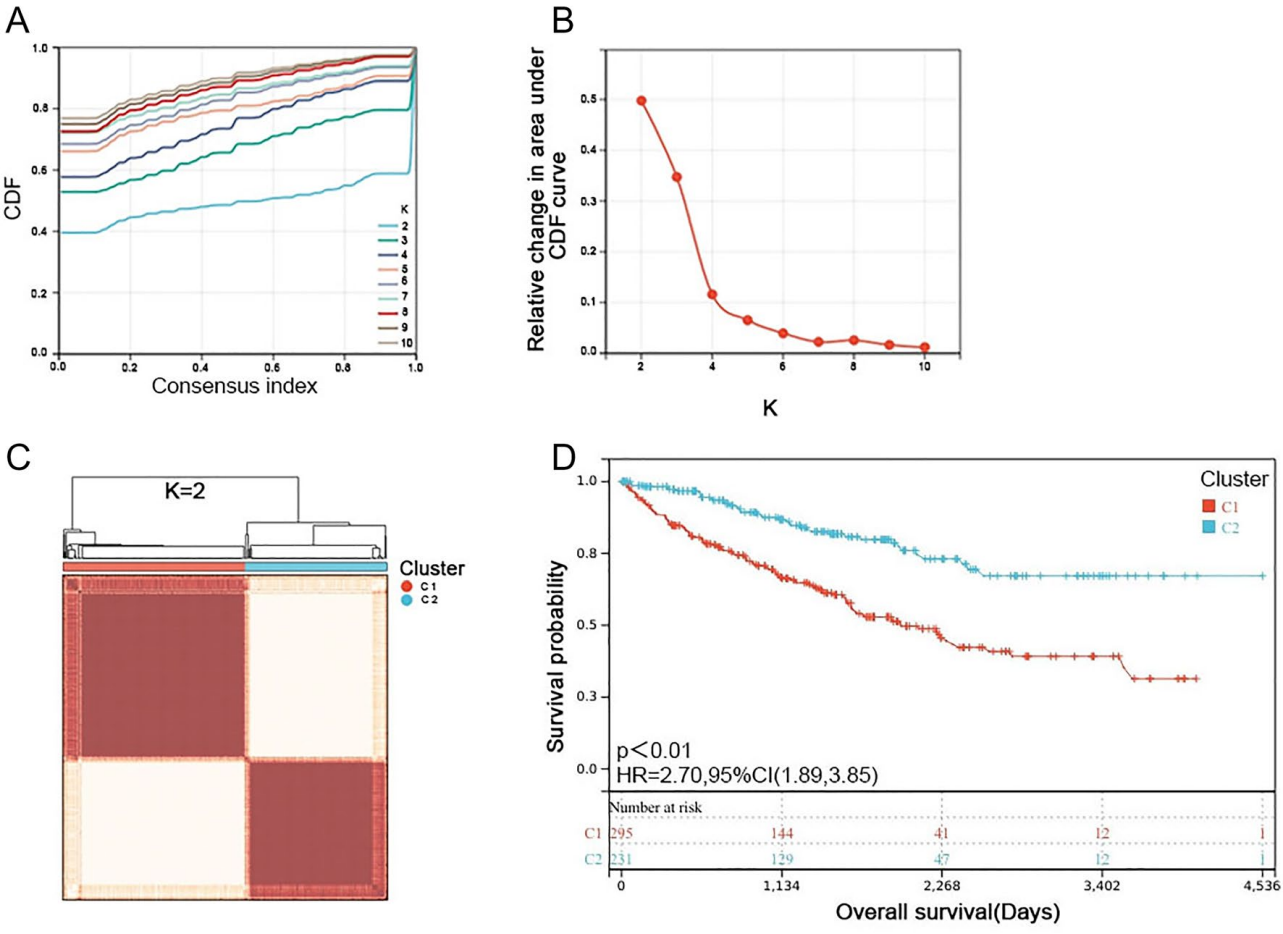
Determination of ICD-associated subgroups by consensus clustering. (**A**, **B**) Delta area curve of the consensus clustering indicates the relative change in area under the cumulative distribution function (CDF) curve for k = 2–10; (**C**) Heatmap exhibits consensus clustering results (k = 2) in 531 ccRCC samples; (**D**) Kaplan–Meier curves of OS in Cluster1 and Cluster2 subgroups.

The k-means algorithm in the "ConsensusCluster" package in R was used to combine the 11 ICD gene expression profiles that were significantly associated with OS for unsupervised clustering. The area under the CDF curve gradually increases when the value of K increases, and we need to keep the area under the curve (AUC) as large as possible, and try to make the CDF Delta fall as slowly as possible. The number of optimal clusters is K = 2, and the number of suboptimal clusters is K = 3 (Fig. 2A,B). The clustering has high stability when the parameter is set to k = 2. The clustering results are shown in Fig. 2C. Statistics of clustering results: C1(N = 298), C2(N = 233).

Therefore, on the basis of clustering, this study divided 531 cancer tissue samples into 2 groups (Cluster1 and Cluster2) and compared the differences between these 2 subgroups. The Kaplan–Meier curves of the two subgroups showed that the overall survival of Cluster 1 was significantly lower than that of the Cluster 2 subgroup (Fig. 2D). We compared the expression of related ICD genes at the mRNA level in these two subgroups and found that FOXP3, IL6 and LY96 were significantly highly expressed in Cluster 1 compared to other genes (Fig. S2).

### PCA found FOXP3 and LY96 play an important role in Cluster 1

To continue to explore the role of different ICD grouping patterns in this prognostic difference and to gain insight into Cluster1, this study used a PCA algorithm to find the key components and major molecules in Cluster1. Figure S3A shows the contribution of each principal component to the grouping, where we find that principal component Dim 1 accounts for 25.6% of the contribution in the grouping and principal component Dim 2 accounts for 21.1% of the contribution in the grouping. Afterwards, we analyzed the two subgroups in the distribution of principal components, as shown in Fig. S3B, and found that it was principal component Dim 1 that mainly distinguished Cluster 1 from the other subgroup Cluster 2. Therefore, we further analyzed the joint share (Fig. S3C) and the separate share of each factor in Dim1 (Fig. S3D). In the process of calculating the contribution of each factor in Dim1, we found that FOXP3, and LY96 have a greater impact on the principal component Dim1, and the main role of these two is to increase the weight of principal component Dim1.

The result of the PCA indicates that, at the level of mRNA expression, the two factors that play an important role in Cluster 1 are FOXP3 and LY96, and this result is in agreement with the results of Fig. S2B.

### WGCNA displayed the turquoise gene module had a close relationship with Cluster 1

The above analysis showed that FOXP3 and LY96 were the main influencing factors in Cluster 1, so these two genes caught our attention. We calculated the correlation of all genes with two genes, FOXP3 and LY96, and considered genes with a p value < 0.05 and Spearman's Correlation ≥ 0.50 as significantly associated. 600 genes were found to be co-expressed with FOXP3 and 463 genes were co-expressed with LY96. The two sets of genes were taken to intersect and plotted on a Venn diagram (Fig. S4). A total of 240 overlapping genes were finally found to exist. These overlapping genes were then incorporated into the WGCNA for analysis. The results of the soft threshold selection during the analysis are displayed in Fig. S5. Selected genes were divided into 3 modules by WGCNA.

We calculated the association between modules and clinical phenotypes using the Spearman correlation analysis method. The highest correlation was found between the turquoise module and Cluster 1, with a correlation coefficient R = 0.51 (p < 0.0001) (Fig. S5D). The genes in the turquoise module may have an influence on the characteristics of Cluster 1.

### Modular gene function enrichment analysis

To further understand the function of the turquoise module gene and thus to investigate the functional characteristics of Cluster 1, we performed the Gene Ontology (GO), Kyoto Encyclopedia of Genes and Genomes (KEGG) functional enrichment analysis and Gene Set Enrichment Analysis (GSEA).

GO enrichment analysis revealed that the enrichment results of these genes were mainly focused on immunity, including activation, infiltration, and regulation of immune cells (Fig. S6A). KEGG analysis showed that Cluster 1 was closely related to primary immunodeficiency, Th17 cell differentiation, and the B cell receptor signaling pathway (Fig. S6B). When GSEA analysis was performed, a minimum gene set of 5 and a maximum gene set of 5000 with 1000 replicate sampling was set based on gene expression profiles and phenotypic groupings, with P value < 0.05 and FDR < 0.1 being considered statistically significant. GSEA analysis found that the cytosolic DNA sensing pathway, which is the trigger of innate immune^34^ was enriched in Cluster 1(Fig. S6C).

### Construction of the predictive model PRE

**Figure 3:**
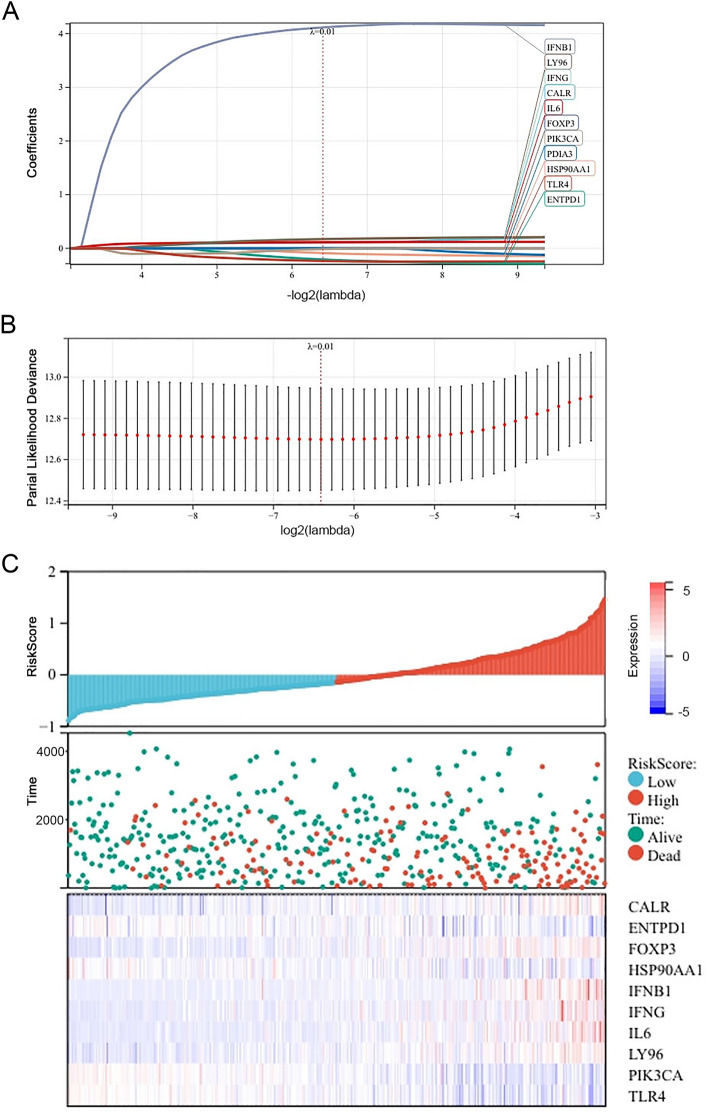
Constructing a predictive signature by LASSO-COX analysis. (**A**, **B**) Identifying 10 ICD genes most associated with OS; (**C**) Risk score curves, vital status distribution of each ccRCC patient in TCGA database, and heatmap of the prognostic 10 ICD genes.

By LASSO-COX dimension reduction analysis (Fig. 3A,B), ten candidate genes (CALR, ENTPD1, FOXP3, HSP90AA1, IFNB1, IFNG, IL6, LY96, PIK3CA, and TLR4) and their corresponding lambda values were used to calculate the risk score for each patient. The risk score model, whose name is "PRE", was developed premised on the formulas below: RiskScore = 0.112*CALR − 0.207*ENTPD1 + 0.001*FOXP3 − 0.088*HSP90AA1 + 4.115*IFNB1 + 0.174*IFNG + 0.114*IL6 + 0.172*LY96 − 0.014*PIK3CA − 0.234*TLR4.

### Predictive value of PRE in prognosis and immunotherapy

**Figure 4:**
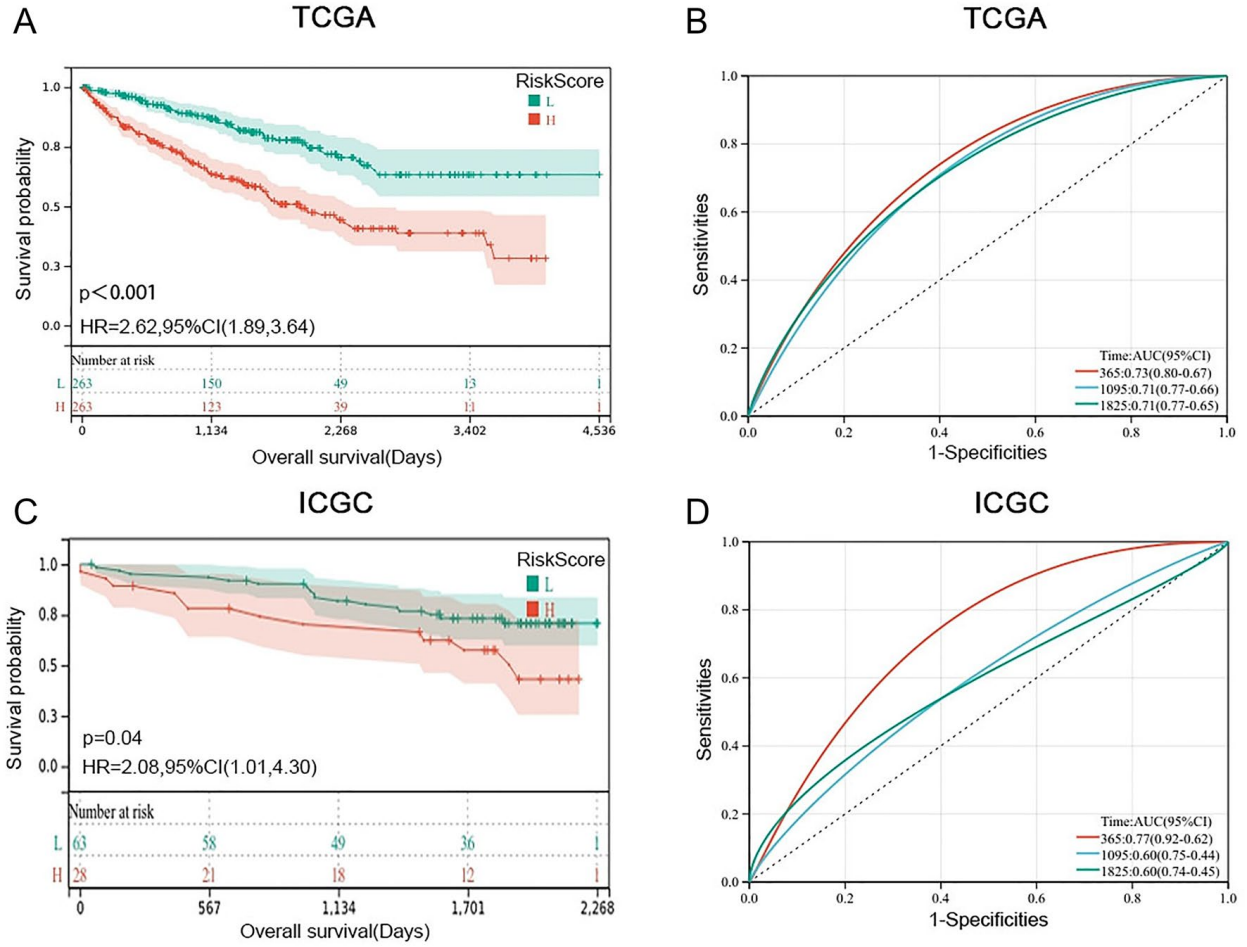
The Kaplan–Meier and ROC curves of the 10-gene signature in predicting the prognosis of ccRCC. (**A**, **C**) The Kaplan–Meier curves of training set and validation set indicated that patients in RiskScore-H group have shorter OS than patients in the RiskScore-L group; (**B**, **D**) The results showed that the 365-,1095-, and 1825-day ROC curves all had an area under the ROC curve (AUC) of greater than 0.6 in the two databases (TCGA and ICGC).

Firstly, we explored the prognostic impact of PRE in ccRCC patients. The median risk score (− 0.14) of the training database was set as the cutoff value (Fig. 3C). Survival analysis indicated that the RiskScore-low group had prolonged survival time in the TCGA cohort (Fig. 4A, p < 0.001), which was further corroborated in the ICGC RECA-EU cohort (Fig. 4C, p < 0.05).

The roc and ci function of the pROC package in R evaluate the AUC and confidence interval of the prediction model PRE at 365-day, 1095-day, and 1825-day time points. We find that its c-index value is around 0.7 in both the training set and the validation set (Fig. 4B,D).

What’s more, our results showed that the number of alive statuses in the low-risk group was much higher than in the high-risk group. Moreover, the heat map of Fig. 3C indicated that ENTPD1, HSP90AA1, PIK3A, and TLR4 decreased with increasing risk score and were prognostic protective factors, while CALR, FOXP3, IFNB1, IFNG, IL6 and LY96 were prognostic dangerous factors.

**Figure 5:**
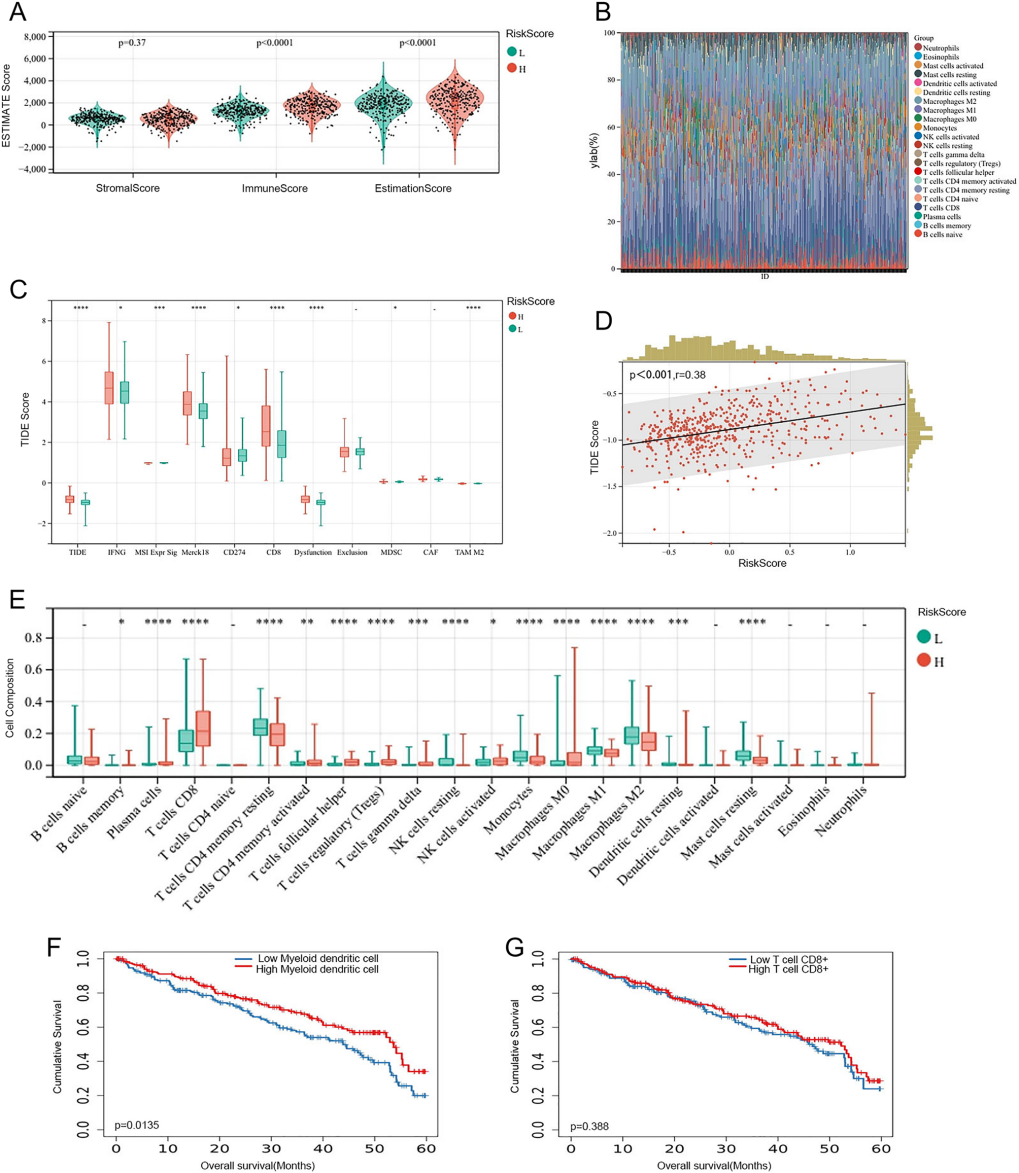
The landscape of immune infiltration in RiskScore-H and RiskScore-L groups. (**A**) For stromal score, immune score, and estimation score, the median and quartile estimations are shown by violin plots; (**B**) Proportion of different immune cells between RiskScore-H group and RiskScore-L group; (**C**) TIDE scores were significantly lower in the low-risk group than that in the high-risk group; (**D**) A positive correlation between TIDE scores and PRE risk scores (p < 0.001, r = 0.38); (**E**) 22 kinds of immune cells between two groups calculated by the CIBERSORT approach; (**F**) Low dendritic cell infiltration in ccRCC implies a poor prognosis (p = 0.0135); (**G**) The degree of CTL infiltration is less important in ccRCC (p = 0.388).

Secondly, we explored if the PRE could assess the immune microenvironment of tumors and predict patient responsiveness to immunotherapy. Accumulating evidence suggests that ICD has a great effect on the activation of certain antitumor immune responses^12,15,16^. In this research, we analyzed the composition of the tumor microenvironment between the RiskScore-high group and the RiskScore-low group. Overall, the immune score and estimate score were higher in the RiskScore-high group compared to the RiskScore-low group (Fig. 5A).

In addition, we assessed differences in immune infiltration of 22 kinds of immune cells between two groups utilizing the CIBERSORT approach. Figure 5B summarises the results obtained from 531 ccRCC patients from the TCGA-KIRC cohort. Patients in the RiskScore-high group, in particular, had significantly lower percentages of resting CD4^+^ T cell memory, resting NK cell, Monocyte, macrophage M1 and M2, resting dendritic cell and resting mast cell (Fig. 5E). This implies poor antigen presentation and immune activation in the Cluster 1 subgroup.

Immunotherapy has demonstrated improved survival in the treatment of multiple tumor types, and it is urgent to identify patients who will benefit most. Thus, we further explored whether PRE could predict the efficacy of immunotherapy through TIDE. In our results, TIDE scores were significantly lower in the low-risk group than that in the high-risk group, implying that patients with low PRE scores could benefit more from immunotherapy (Fig. 5C). When scatter correlation analysis was performed between TIDE scores and PRE risk scores, we found a positive correlation (p < 0.0001, r = 0.38), indicating that as risk score increases, patients have a greater probability of developing immune resistance (Fig. 5D).

Interestingly, we found that the proportion of CD8^+^ T cells was significantly higher in the high-risk group than that in the low-risk group (Fig. 5C, p < 0.0001), which is similar to that of the CIBORSORT analysis.

### Nomogram can accurately and stably predict OS

**Figure 6:**
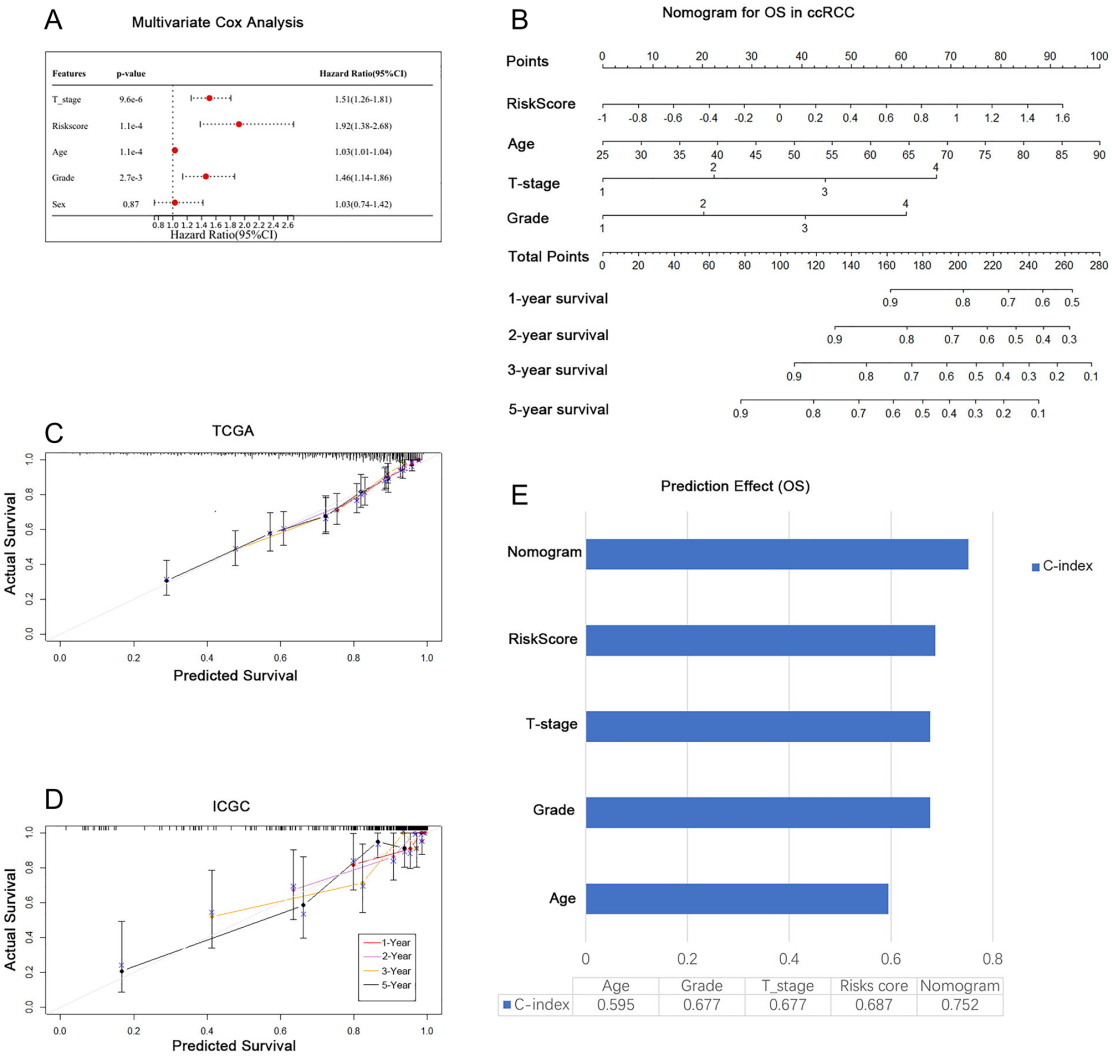
The nomogram predicts the OS accurately and stably in ccRCC. (**A**) Multivariate Cox analysis identified the independent predictors of survival status; (**B**) The 1-, 2- 3-, and 5-year deceased rate of ccRCC patients could exactly be predicted by the nomogram; (**C**, **D**) The Calibration plots showed the comparison between predicted and actual OS in training and validation sets; (**E**) The final nomogram has the highest predictive power (C-index = 0.752).

To facilitate the clinical application of the prognostic prediction model, an individualized prediction model for OS was constructed. The results of the multivariate COX analysis (Fig. 6A) show that T-stage, age, grade, and the risk score of the PRE model were independent predictors of survival status and the individualized prediction model for OS was premised on them. In the nomogram, the top part is the scoring system, and the bottom part is the prediction system. Total points, the sum points of each factor, could exactly predict the 1-, 2-, 3-, and 5-year survival rates of ccRCC patients (Fig. 6B). Figure 6C,D exhibited that the predicted and actual OS in the calibration curve had a satisfactory overlap in training and validation cohorts, indicating an optimal predictive accuracy. C-Index values were used to show the accuracy of each individual predictive factor in the survival prediction, and the c-index of this nomogram model was 0.752, which is higher than any other predictive factor (Fig. 6E).

### Potential therapeutic compounds

Connectivity map (CMAP) analysis was used to explore the association between genes, chemicals, and compounds to search for candidate drugs that might target pathways associated with ICD genes.

Finally, we found 54 candidate inhibitors by the SPIEDw tool and 30,973 possible target chemicals by the Broad Institute’s Connectivity Map build 02 tool. The two have 41 overlapping inhibitors and are ranked according to the connectivity score. Table 1 shows the top 10 drugs with the highest connectivity score, together with the target and mechanism of action (MOA) of these drugs.

**Table 1:** Ten potential compounds from the CMAP with the targets and mechanisms of action (MOA) of these drugs.

Compound	Cell name	MOA	Target
Alprostadil	PC3	Prostanoid receptor agonist	PTGER1/PTGER2/CATSPER1/CATSPER2/CATSPER3/CATSPER4/PTGDR/PTGER4/PTGIR
MK-1775	NCIH596	WEE1 kinase inhibitor	WEE1
Josamycin	YAPC	Bacterial 50S ribosomal subunit inhibitor	NA
Diphenhydramine	VCAP	Histamine receptor antagonist	HRH1
Tolazamide	SKB	ATP channel blocker	ABCC8/KCNJ1/KCNJ10/KCNJ11
Scoulerine	PHH	1. Adrenergic receptor antagonist 2. GABA receptor antagonist 3. Serotonin receptor antagonist	ADRA1D/ADRA2A/GABRA1
Lopromide	MCF7	Radiopaque medium	NA
Metronidazole	HEPG2	Bacterial DNA inhibitor	CYP2C8/CYP2E1/CYP3A5/CYP3A7/CYP2C9/CYP3A4
Racecadotril	HA1E	Enkephalinase inhibitor	MME
Radicicol	SKBR3	NA	NA

*MOA* mechanism of action, *NA* not acquired.

## Discussion

After ID transformation, normalization, and deletion of highly missing sample data in the raw expression data, a total of 603 TCGA-KIRC samples, including 531 cancer samples and 72 normal control samples, were finally included in the training set of this study, and a total of 136 ICGC samples were included in the validation set. In this study, a predictive model based on ICD-related genes, PRE, was developed to accurately and stably assess the tumor microenvironment and its sensitivity to immunotherapy (Fig. 5C), thus further predicting patient prognosis. Furthermore, in order to improve the clinical applicability of the prediction model, we also combined clinicopathological factors and PRE to create a nomogram for predicting OS in ccRCC patients. In contrast to previous studies^35^, we also included T-stage as an independent risk factor in the nomogram. Validation of the calibration curves in the training and validation sets proves that our nomogram has a robust predictive accuracy (Fig. 6). In the nomogram, the higher the patient's risk score, T-stage, grade, and age, the lower the 1-, 2-, 3-, 5-year survival rate. The C-index, also known as the concordance index, can be used to estimate the probability that the predicted outcome of an event will be the same as the actual outcome. The C-index value indicates that the final nomogram has the highest predictive power (C-index = 0.752), higher than any of the other independent predictors (Fig. 6E).

When Kaplan–Meier survival analysis was performed, five cancer samples were excluded because no survival data were available (Fig. 2D). GO and KEGG analysis revealed that related gene functions were mainly enriched in primary immunodeficiency and Th cell differentiation, suggesting that the biological progression of ccRCC may be related to the inability of the initial immune response, a result also reported in a previous study by Gong et al.^36^. GSEA analysis found that cytosolic DNA sensing pathway which is the trigger of innate immune^34^ was enriched in Cluster1. In a nutshell, results of the Gene Ontology (GO) and Kyoto Encyclopedia of Genes and Genomes (KEGG) functional enrichment analysis and Gene Set Enrichment Analysis (GSEA) showed the close relationship between Cluster1-related genes and regulation and activation of the immune system.

**Figure 7:**
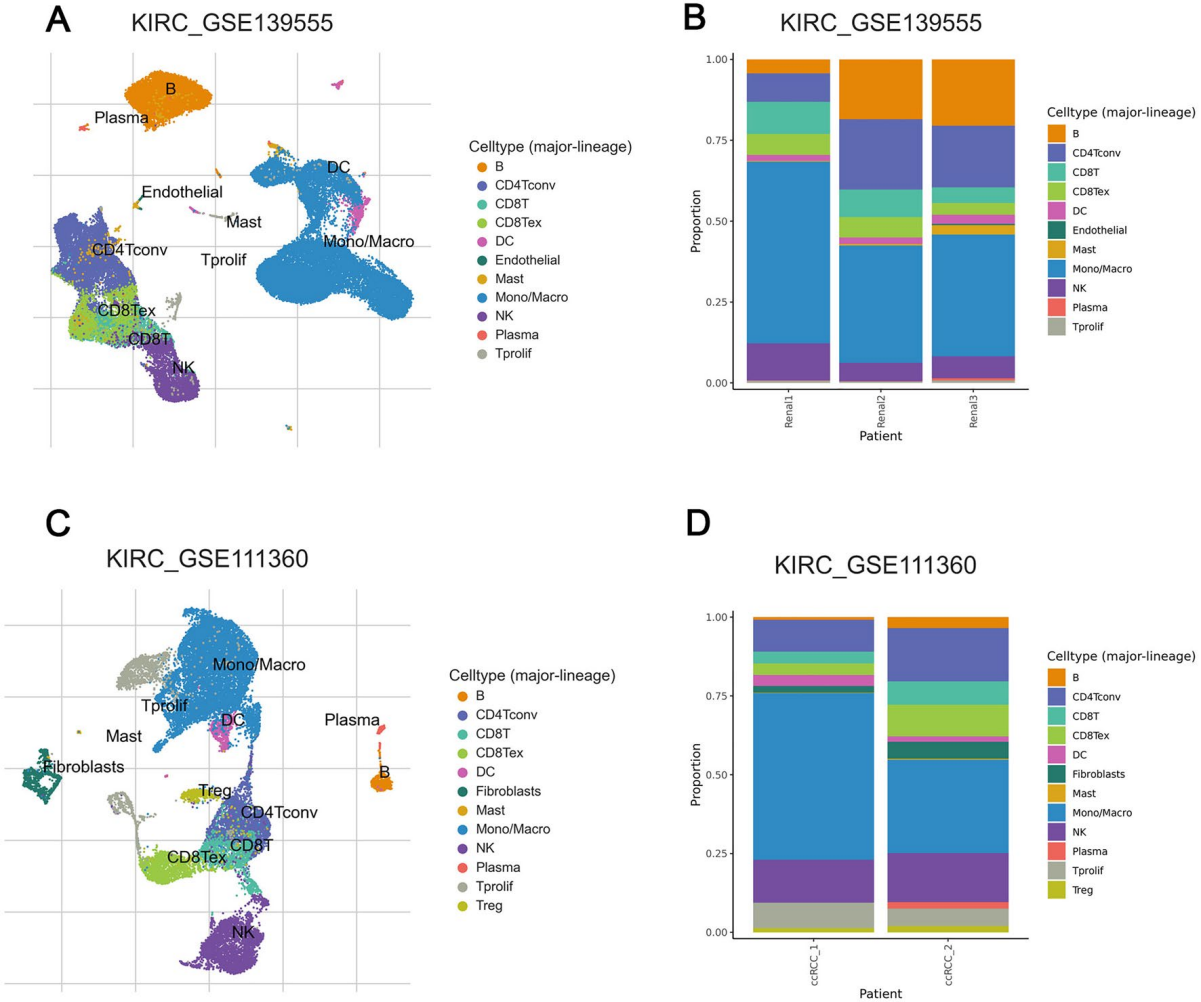
Single cell sequencing results of immune cells shows the low proportion of DC (dendritic cell) in GSE139555 (**A**, **B**) and in GSE111360 (**C**, **D**).

Interestingly, the ESTIMATE results found that the RiskScore-H group had a higher immune score and estimate score, a result similar to the findings of Wang et al.^20^. Combined with the CIBERSORT (Fig. 5E) results, we found that the proportion of killer cells such as NK cells or CD8^+^ T cells was not low in kidney cancer. But due to the lack of APC cells, this resulted in the inability of antigen presentation and immune activation, which corroborates the previous results of GO and KEGG gene enrichment analysis. Meanwhile, the TIMER2.O (Fig. 5F,G) and TISCH (Fig. 7) results demonstrate that low dendritic cell infiltration in ccRCC implies a poor prognosis, whereas the degree of CTL infiltration in ccRCC is less important, which is a strong argument for our previous findings. Lastly, we found that the RiskScore-H group scored significantly higher than the group of RiskScore-L on dysfunction and exclusion in TIDE results, suggesting that it may be due to T cell dysfunction or immune escape that CTL could not play its original role of tumor cell killing.

There are some problems and limitations of this study that need to be explained. This is a retrospective study, and selection bias is inevitable. Prospective clinical studies in follow-up studies may help to further determine the accuracy of the nomogram model. We found that the ten candidate ICD genes (CALR, ENTPD1, FOXP3, HSP90AA1, IFNB1, IFNG, IL6, LY96, PIK3CA, and TLR4) may affect the prognosis of ccRCC, but their mechanisms of action have yet to be studied in vitro and in vivo, and we are also working on related basic experiments to investigate the mechanisms and possible biological functions. Novel therapeutics based on the activation of cell death-related genes for the treatment of carcinoma have achieved the new progression^37^. The inhibitory effects of these potential compounds provided in this study have been validated in basic studies of other cancer cell lines such as PC3, but have not been reported in kidney cancer cell lines. Therefore, further basic and clinical validation of the inhibitory effects of these target compounds in RCC is needed.

## Conclusion

Our study used univariate COX analysis, multivariate COX analysis, and Lasso-Cox regression to screen variables. A predictive model, PRE, was constructed to both assess the pattern of immune cell infiltration in ccRCC and predict the responsiveness of patients to immunotherapy, as well as predict the probability of long-term survival. The PRE model constructed based on the ICD-related genes was further combined with clinical factors to improve the accuracy and applicability of the model. Finally, candidate target drugs with potential therapeutic effects are also provided.

## Supplementary Information

Supplementary Information.

## Data Availability

The original contributions presented in the study are included in the article/supplementary material, further inquiries can be directed to the corresponding author. The data generated by the TCGA Research Network (https://www.cancer.gov/tcga) and the ICGC Research Network (https://dcc.icgc.org) served as the basis for the findings published here.
