# Supplementary material for: Identification of an immunogenic cell death-related gene signature predicts survival and sensitivity to immunotherapy in clear cell renal carcinoma
Source: Sci Rep. 2023 Mar 17;13:4449. doi: 10.1038/s41598-023-31493-z (PMC10023707; doi:10.1038/s41598-023-31493-z)
Supplement: Supplementary file 1 — Supplementary Information. [file 41598_2023_31493_MOESM1_ESM.zip › Supplementary material/Figures/Figure legends.docx]

**Figure S1** Univariate Cox analysis filters the ICD genes significantly associated with overall survival (OS).

**Figure S2** Distinct expression of ICD genes in Cluster1 and Cluster2. (A) Heatmap of 34 ICD-related genes expressions in Cluster1 and Cluster2; (B) Box plots present different expression of ICD genes between Cluster1 and Cliuster2.

**Figure S3** Results of principal component analysis. (A) Contribution values of each principal component to the subgroups; (B) The relationship between principal components and subgroups is shown; (C) The proportion of independent factors contributing in the principal components; (D) Contribution percentage of each factor in principal component 1.

**Figure S4** Venn diagram shows the genes co-expressed with FOXP3 and LY96.

**Figure S5** Results of weighted gene co-expression network analysis (WGCNA). (A, B) WGCNA screens for soft threshold; (C) Screening the gene module co-expressed with FOXP3 and LY96; (D) The heatmap shows the correlation between gene modules and cluster groupings.

**Figure S6** Gene function enrichment analysis. (A) GO enrichment analysis reveals that the enrichment results of these genes were mainly focused on immunity; (B, C) KEGG and GSEA analysis show that Cluster 1 was closely related to primary immunodeficiency.
