# Supplementary figures and images for: Identification of an immunogenic cell death-related gene signature predicts survival and sensitivity to immunotherapy in clear cell renal carcinoma
Source: Sci Rep. 2023 Mar 17;13:4449. doi: 10.1038/s41598-023-31493-z (PMC10023707; doi:10.1038/s41598-023-31493-z)

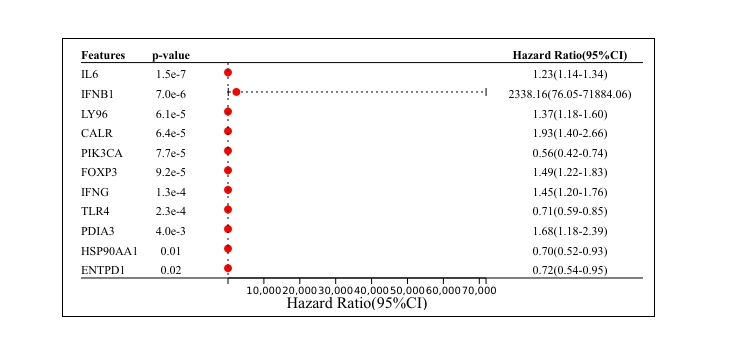

Supplement: Supplementary file 1 — Supplementary Information. [file 41598_2023_31493_MOESM1_ESM.zip › Supplementary material/Figures/Figure S1.jpg]

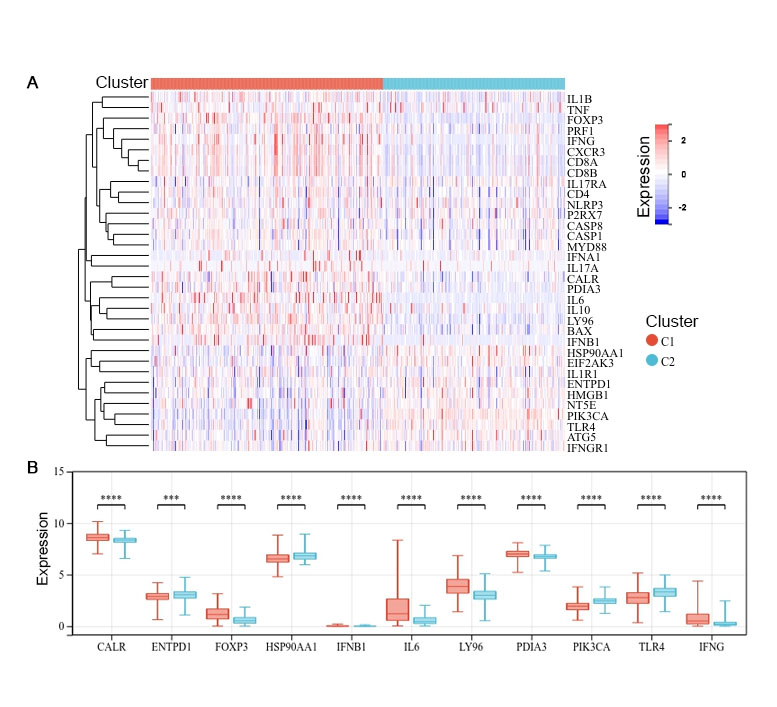

Supplement: Supplementary file 1 — Supplementary Information. [file 41598_2023_31493_MOESM1_ESM.zip › Supplementary material/Figures/Figure S2.jpg]

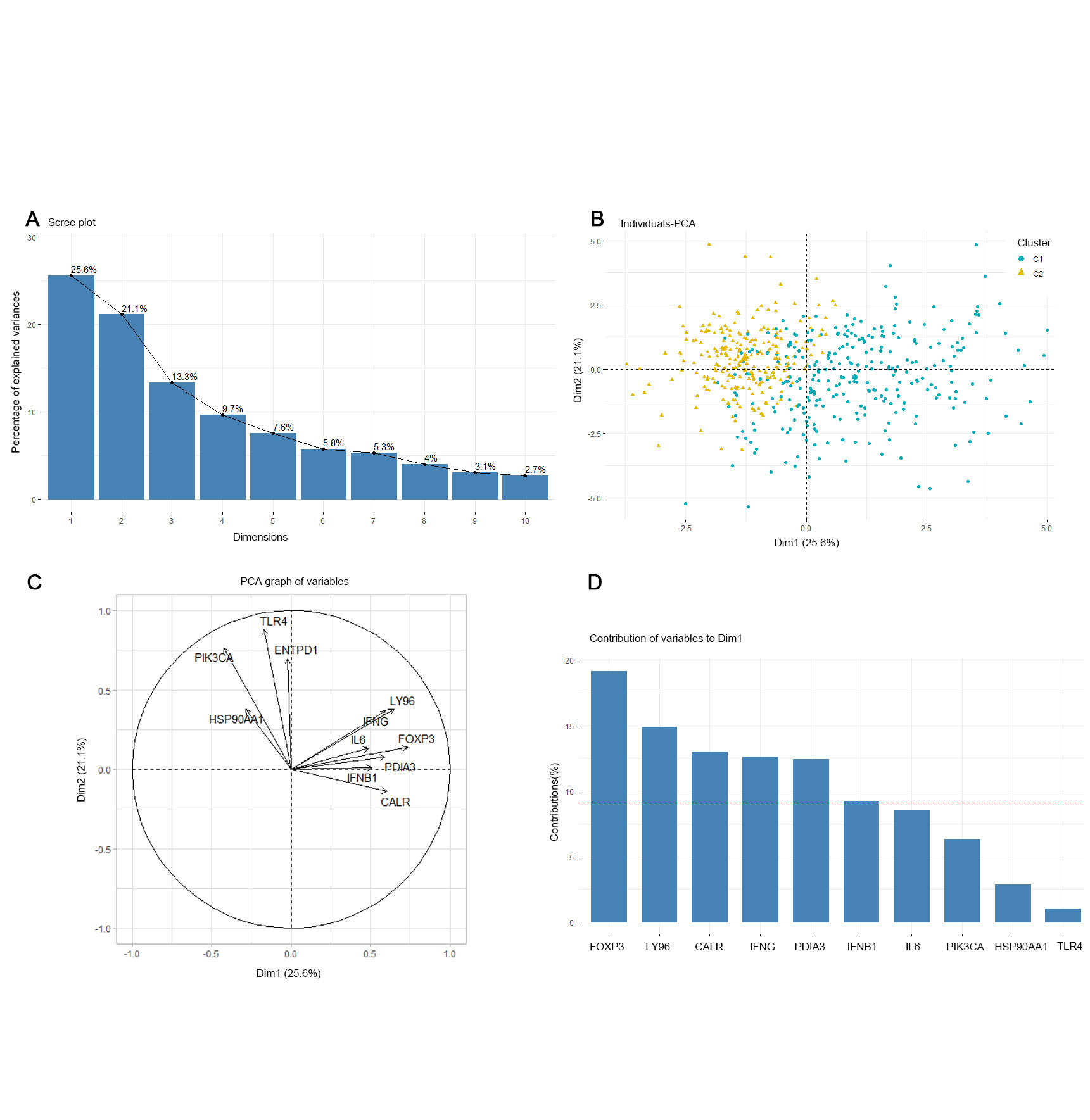

Supplement: Supplementary file 1 — Supplementary Information. [file 41598_2023_31493_MOESM1_ESM.zip › Supplementary material/Figures/Figure S3.jpg]

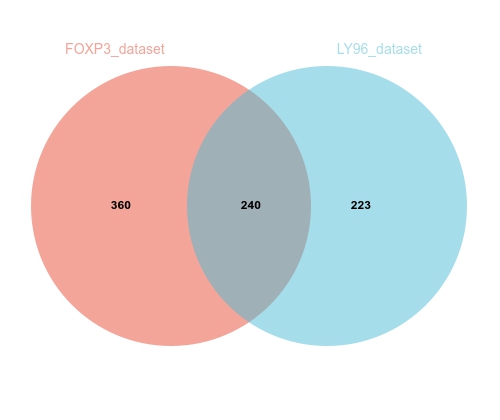

Supplement: Supplementary file 1 — Supplementary Information. [file 41598_2023_31493_MOESM1_ESM.zip › Supplementary material/Figures/Figure S4.jpeg]

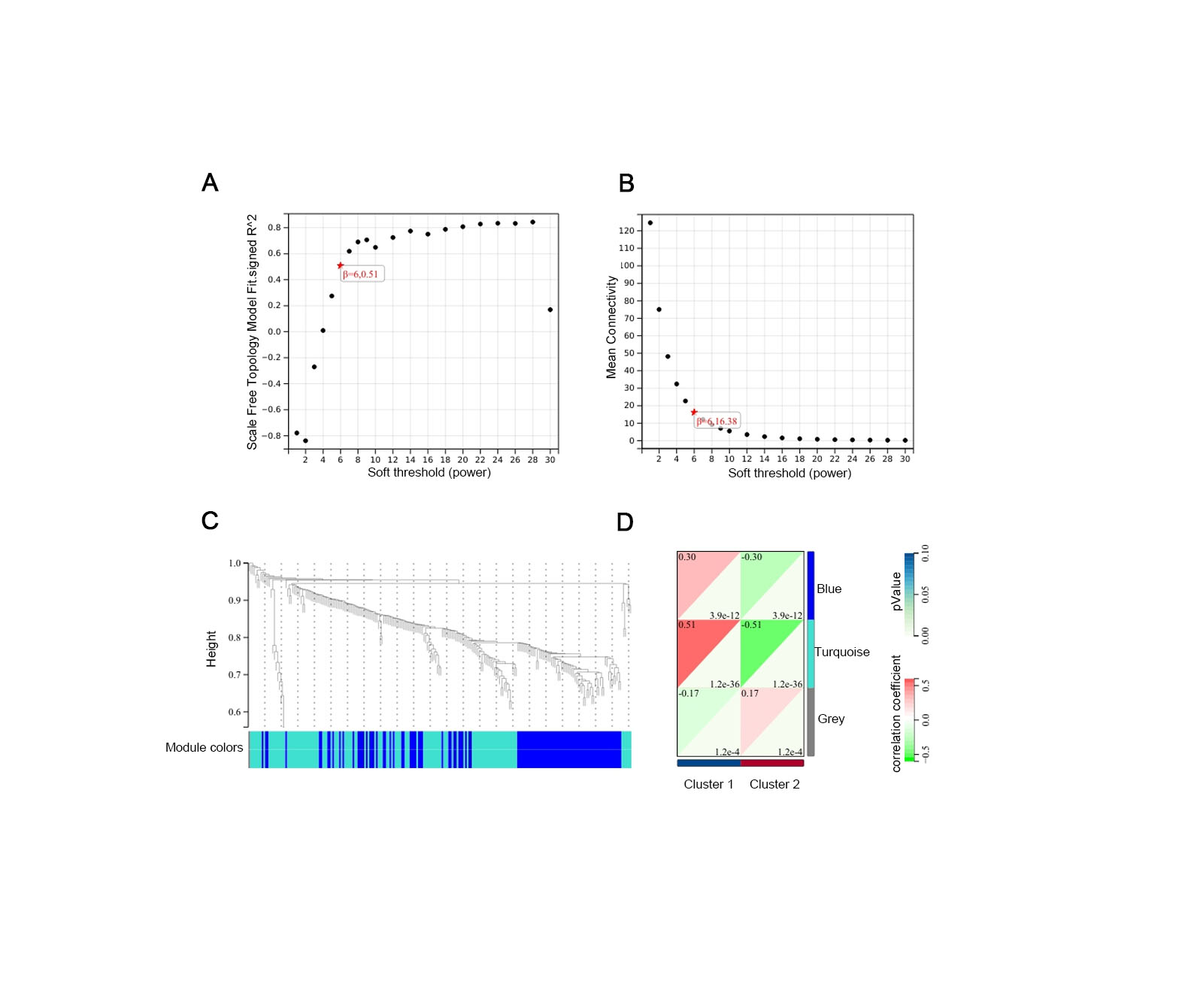

Supplement: Supplementary file 1 — Supplementary Information. [file 41598_2023_31493_MOESM1_ESM.zip › Supplementary material/Figures/Figure S5.jpg]

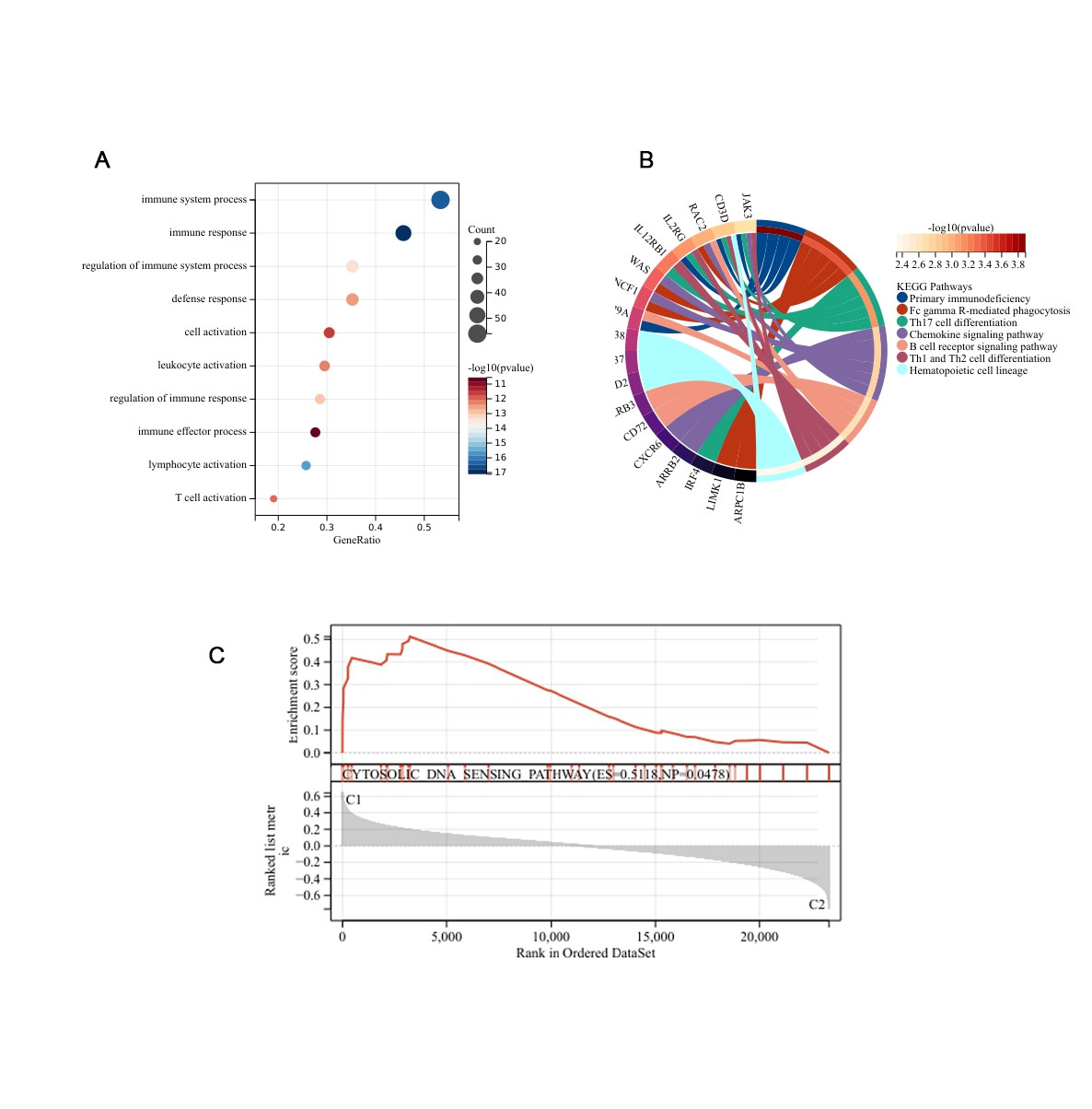

Supplement: Supplementary file 1 — Supplementary Information. [file 41598_2023_31493_MOESM1_ESM.zip › Supplementary material/Figures/Figure S6.jpg]
